# Computer Kinesiology: New Diagnostic and Therapeutic Tool for Lower Back Pain Treatment (Pilot Study)

**DOI:** 10.1155/2020/2987696

**Published:** 2020-08-24

**Authors:** Pavla Honcu, Petr Zach, Jana Mrzilkova, Dobroslava Jandova, Vladimir Musil, Alexander Martin Celko

**Affiliations:** ^1^Department of Rehabilitation Medicine, Third Faculty of Medicine, Charles University, Prague, Czech Republic; ^2^Department of Anatomy, Third Faculty of Medicine, Charles University, Prague, Czech Republic; ^3^Institute of Physiotherapy and Selected Medical Disciplines, Faculty of Health and Social Studies, University of South Bohemia in České Budějovice, České Budějovice, Czech Republic; ^4^MediCentrum JONA Ltd., Prague, Czech Republic; ^5^Department of Rehabilitation Medicine, University Hospital Kralovske Vinohrady, Prague, Czech Republic; ^6^Centre of Scientific Information, Third Faculty of Medicine, Charles University, Prague, Czech Republic; ^7^Department of Epidemiology and Biostatistics, Third Faculty of Medicine, Charles University, Prague, Czech Republic

## Abstract

The aim of this study was to demonstrate the effectiveness of the diagnostic and therapeutic medical information system Computer Kinesiology in physiotherapy in patients with low back pain who were not responding to conventional therapy. Computer Kinesiology is primarily intended for the diagnostics and therapy of functional disorders of the locomotor system. This pilot study population included 55 patients (Group 1) with acute and chronic back pain and 51 persons (Group 2) without back pain. The third group was a control group of 67 healthy volunteers with no evidence of musculoskeletal pathologies and no back pain. All 173 subjects were examined three times by the diagnostic part of the Computer Kinesiology method. Groups 1 and 2 were treated after every diagnostics. Group 3 was not treated. The effect was evaluated by H score. Improvements after therapy were defined by reducing the H score by at least 1 point. In Group 1, the H score decreased by at least 1 point in 87.3% (95% CI: 75.5-94.7) and in Group 2 in 78.4% (95% CI: 64.7-88.7). There was no change of distribution of H Score grade in Group 3. The improvement neither depended on gender, age, and BMI nor was it influenced by the length of the therapy. This study demonstrated a high therapeutic efficacy of the Computer Kinesiology system in patients with back pain (Group 1) and in persons without back pain (Group 2) who used the Computer Kinesiology system for primary and secondary prevention of back pain.

## 1. Introduction

The issue of back pain is currently a worldwide epidemic. In the United States of America, up to 100 million adults suffer from chronic back pain and their care including work incapacity and disability costs 635 billion US dollars per year [1]. Traditionally, back pain can be divided into an acute, subacute, and chronic. Nevertheless, epidemiological data show that back pain has usually a recurrent, intermittent, episodic character [2].

Prevalence and incidence of the difficulties are as follows: 50-80% of adults have their own experience with back pain during their life, and 40% of the population suffer from back pain once a year. Back pain is one of the most frequent reasons for work incapacity. Currently, back pain appears also in 12% of adolescents at the age of 11. At the age of 15, the number increases up to 50% [2].

In medicine, back pain is referred to by different names or diagnoses such as vertebrogenic algic syndrome, lumbago, sciatica, and degenerative changes of spine. The disease may have motor, sensitive, and vegetative symptoms. The stage of the disease is crucial for every condition. Acute pain lasts up to four weeks, subacute pain lasts for 4-12 weeks, and chronic pain lasts for more than 12 weeks [1, 2]. The latest recommendation of the NIH Task Force on Chronic Back Pain Standard 2015 suggests that after 40 years of examining the causes of LBP (low back pain), the diagnosis according to pathophysiological and/or pathoanatomic criteria are not beneficial [1].

In an acute phase, an accurate diagnosis can be made in less than 15% of patients. This means that 85% of back pain in the past was usually treated with nonspecific care. Simultaneous division of back pain into three groups seems sufficient. These are severe spinal pathologies, nerve root problems, and mechanical back pain—this include 85% of the patients with nonspecific back pain (©The McKenzie Institute International). Thanks to precise subjective and objective examination, an educated physiotherapist is able to distinguish this nonspecific back pain more precisely. Specification is easier thanks to the so-called centralization phenomenon which appears in 70% of acute patients and in 50% of chronic patients with LBP. The use of the centralization phenomenon for the determination of the preferred direction of movement in the therapy testifies to the quality of the care. Terminologically, *centralization phenomenon* is defined as migration of the pain from a distal segment into a proximal one, while *centralization* is defined as a complete removal of peripheral radicular pain as a reaction to applied therapy (position, movements, and mobilization) [3–11].

Conservative treatment of back pain can take a variety of forms, from therapy procedures of spinal manipulative therapy [12, 13], motor control exercises [14], and stabilization exercises [15] to directional preference therapy based on the abovementioned centralization phenomenon, which is a basis of McKenzie's therapy. Classification of patients according to McKenzie's system is based on motion analysis, identification of centralization, directional preference, and mechanical diagnosis [16–19]. If a physiotherapist suspects structural causes of LBP, it is necessary to refer the patient to a consultant or another specialist. Functional blockades of segments [20], muscle imbalance, postural problems, and other problem subgroups are the most frequent problems with the vertebral column [21–23]. Preference of the stabilization exercises against other therapies is not fully justified because their higher efficiency has not been demonstrated [24, 25].

Given the increasing incidence of back pain, the following general recommendations should be taken as a form of prevention: reduction of hypomobility and diversion from passive treatment methods to a patient's motivation to participate actively in prevention and treatment programs, including maintenance of daily regimen measures (drinking regimen, weight maintenance or weight reduction, and sleep patterns, as well as ergonomics of daily activities and work processes).

The aim of the current approach to address LBP growth is primary prevention and secondary prevention of back pain. Primary prevention (educational programs) is focused mainly on endangered adolescents, risk groups with postural difficulties associated with one-sided work and sport load. Secondary prevention is focused mainly on patients with recurrent back pain.

The basic characteristics of the physiotherapy discipline consist in focusing on diagnostics, treatment, and prevention of functional disorders of the motor apparatus and prevention of the development of structural disorders. Physiotherapy uses two basic principles: a good functional form of an organ and long-term disorders of a function (dysfunction) may lead to structural disorders [26–28]. In the current era of evidence-based medicine (EBM), it is necessary in physiotherapy to cope with the critical opinions of clinical medical disciplines towards subjective evaluation of disorders of the motor system functions. These facts are the prompt for searching the options of objectivization and quantification of reflex changes in the locomotor system soft tissues.

Currently, Medical Expert Information System Computer Kinesiology (MEIS CK) seems to be a new and suitable therapeutic aid for the treatment of low back pain (described in our study) that is not responding to conventional therapy (e.g., hospitalization in departments like neurology, orthopedics, and rehabilitation; complex pharmacotherapy treatment; infusion therapy; local injection under CT control; contraindications for operations; and patient's preference of conservative treatment). Primarily, this system is used for the therapy of functional locomotor disorders. Medical MEIS CK meets in a validated manner the EBM requirements for diagnostics of the motor apparatus. MEIS CK supports LBP NICE guidelines for a patient's autotherapy and individual workout [29].

Evaluation of functions in the locomotor system results in numerical outputs and graphs. The procedure of data collection in the MEIS CK system includes examination of a patient mostly in a postural load, but a part of the examinations of lower limbs is performed in a supine position on a bed. Diagnostics include 46 standard physiotherapeutic tests in total (23 on the right and 23 on the left), out of which 10 are active movements, 16 are passive movements with the limbs [30], and 20 examinations are focused on soft tissues (HAZ and trigger points) (for a more detailed description, see Materials and Methods) [31, 32]. Results of examinations were written manually in a computer application as three grades, and each test was graded with one grade (A = normal function; B = less than 50% dysfunction (slight dysfunction); or C = more than 50% dysfunction (severe dysfunction) (significant limitation of movement extent, wrong moving stereotype or inability to perform a movement, or significant changes in soft tissues). After entering all values of the tests (*n* = 46) into a computer, these were processed by a mathematical model included in the software program of Computer Kinesiology. The outputs of the analyses were represented by numerical values and graphs interpreting the number of reflexes coupled with a single spinal segment (the so-called horizontal concatenation of dysfunctions) and at the same time by the movement of muscular chains (the so-called vertical concatenation of dysfunctions) [31–36]. Based on the diagnostic part output, the MEIS CK system suggests therapy by manual correction (massage—performed by a trained physiotherapist) and an individual combination of daily home exercises for the patient.

In 2001, teaching MEIS CK in the postdoctoral study of physiotherapists and physicians in the Institute for Postgraduate Medical Education (Prague, Brno) was approved by the Czech Medical Chamber. MEIS CK is suitable for locomotor system dysfunction objectivization and statistical data processing. It is also used for the evaluation of spa therapy effect in spa Bohdaneč, Františkovy Lázně, Jeseník, Košumberk, Luhačovice, Mariánské Lázně, and Slatinice. In the long term, repeated examinations could be used for the evaluation of treatment progress/regress [37]. In 2017, this was done in the spa Jeseník study evaluating the effect of spa rehabilitation care. In this study, in order to evaluate our findings before and after treatment, we used a diagnostic part of MEIS CK. The results of CK diagnostics were correlated to neurohormonal levels and to Knobloch N5 questionnaire results [38–41].

The aim of this study was to demonstrate the effectiveness of the diagnostic and therapeutic Medical Expert Information System Computer Kinesiology in physiotherapy in patients with low back pain who were not responding to conventional therapy. This was carried out in two selected groups, i.e., patients with acute and chronic back pain and healthy participants without back pain. It is the first study that evaluates the effectiveness of the MEIS CK therapy in low back pain.

## 2. Materials and Methods

### 2.1. Characteristics of the File

The study was realized during years 2014-2018. The Ethics Committee of the Third Faculty of Medicine approved the study, and written informed consent was obtained from all participants (no. 20190001/H/1). The patients with acute or chronic back pain who were previously treated by physicians and physiotherapists without any effect (reported in the patient's medical history) and who actively sought a Computer Kinesiology (CK) workplace were placed in Group 1. The persons without back pain, who chose MEIS CK for primary prevention of musculoskeletal disorders and actively sought a CK workplace were included in Group 2. The control group, Group 3, was composed of healthy volunteers who actively sought a CK workplace. All persons were from the Czech Republic population.

A total number of 173 participants (57 men and 116 women) in this study was divided into three groups. Group 1 consisted of 55 patients with acute and chronic back pain who were treated with MEIS CK. From this group, 23 patients suffered from radicular syndrome unilaterally in the lower limbs, and 17 had evidence of intervertebral disc prolapse on MRI or CT. In Group 1, there were 24 men and 31 women; the average age was 43.7 ± 9.7; BMI was 26.4 ± 4.5; and the duration of therapy/follow-up was 70.5 ± 51.7days. The participants from Group 2 (51 persons) chose MEIS CK for primary prevention of LBP and were treated by it. In Group 2, there were 12 men and 39 women; the average age was 47.4 ± 13.5; BMI was 25.6 ± 5.3; and the duration of therapy/follow-up was 140.9 ± 90.3 days. Group 3 consisted of 67 healthy volunteers (control group) who were not treated during this study. In Group 3, there were 21 men and 46 women; the average age was 36.5 ± 15.7; BMI was 25.0 ± 4.5; and the duration of follow-up 99.8 ± 65.4 days (Table 1).

Exclusion criteria for the participation in the study covered the following spinal conditions: infection, fracture, tumor, cauda equina syndrome, spinal cord compression and/or inflammatory disease, pregnancy, history of stroke or infarction, and multiple sclerosis. Anamnesis of all participants was acquired during the first entrance examination by a physiotherapist. Subjective assessment of the efficacy of the treatment by VAS (Visual Analogue Scale) was not performed because it could not be applied in Groups 2 and 3 (there was no LBP).

### 2.2. Medical Expert Information System Computer Kinesiology (MEIS CK)

MEIS CK is primarily designed for the diagnostics and design of the treatment of functional disorders of the musculoskeletal system while respecting the fact that each person has its own individual norm.

The organism responds integrally to external and internal stimuli. MEIS CK depicts current disorders not only of the locomotor system itself but also reflective and segmental projection and associated distant reflex symptoms of visceral organ disorders and motor system response and metabolic response to endocrine disorders and psychosomatic effects [27, 42, 43].

MEIS CK consists of a diagnostic part and a therapeutic part. The diagnostic part was performed in all of the three groups by means of specialized tests (further on referred to as tests). The individualized therapeutic part was done only in Groups 1 and 2 according to CK principles.

#### 2.2.1. Diagnostic Part

Numerical and graphical outputs of the diagnostic section served in designing the treatment process and checking its effectiveness. The decisive indicator of the effect of treatment is the dynamics of changes in the monitored parameters. CK diagnostics use 46 standard physiotherapy tests: ten tests of active movements (head rotation in the eye horizontal plane without head tilting or lateroflexion, trunk of body lateroflexion, and abduction and elevation of an extended upper limb—for each separately), 16 tests of passive movements (hip joint flexion with knee joint bend, hip joint flexion with extended knee joint, hip joint abduction, hip joint adduction, foot plantar flexion, foot dorsal flexion, knee joint flexion, and hip joint extension) [44], and 20 tests of palpation of reflexive changes—trigger points in selected muscles (flexor digitorum profundus, deltoid, pectoralis major, transverse part of trapezius, ascending part of trapezius, erector spinae, gluteus maximus, gracilis, iliotibial tract, and soleus) [27, 31, 32]. Each test was assessed by 3 grades: A, B, or C (such as the shortened muscle tests by Janda) [45]. Grade A is normal function, grade B is a slight dysfunction, and grade C is severe dysfunction. Evaluation of active and passive movements corresponds to Cyriax and Cyriax [46] and Kendall et al. [47]: A—range of movement is normal; B—range of movement is limited till 50%; and C—range of movement is limited by more than 50%. Similarly, we evaluated the findings of the trigger points (TP) in the muscles: A—no TP; B—muscle belly increased tension; C—presence of increased muscle belly tension and TP. Evaluation of TP palpation corresponds to examination rules by Travell, Simons, and Simons [48].

Each test is processed by a mathematical model included in the software program of Computer Kinesiology, version *Profi Complex Start 14.1*. Values were determined experimentally in correlation with spinal cord segment reflex projections and myofascial chains. The numerical outputs and graphs of the CK subroutines could thus provide information about the peaks of the faults of the locomotor system. The CK test section helps to find parts of the locomotor system that deviate the most from the physiological norm in their functions, and the diagnostic part helps to detect early functional disorders. Values of individual limits were determined experimentally. The diagnostic part of the MEIS CK system both provides the summary of numerical values of the motor system dysfunction as a whole, and predicates dysfunctions of body systems and organs. The MEIS CK system does not replace auxiliary medical examinations (laboratory tests, EMG, X-ray, CT, MRI, etc.) or classical medical differential diagnostics of organ pathology. The result of the software processing of numbers and graphs could help to consider potential causes of functional disorders of the motor system in the area of biomechanics, internal medicine, disorders of movement control, and psychosomatic causes [35].

#### 2.2.2. Therapeutic Part

Therapy in Groups 1 and 2 always included two parts—a manual correction of reflexive changes of soft tissues according to MEIS CK on the day of diagnostics and an individual set of exercises with a special breathing regime according to MEIS CK performed by a participant twice a day (till the next check-up—selected breathing regime was *second's rhythm*, i.e., inspiration through the nose for 3 seconds, 2-second pause, expiration through the nose for 4 seconds, and 2-second pause. The breathing cycle was repeated 3-6 times in each position. The reason for the use of breathing exercises comes from the physiology of the brain stem reticular formation. Briefly, inspirium facilitates activity so that muscle tonus increases, while expirium facilitates an inhibitory effect on muscle tonus. This is a relatively safe method compared to other movements and could be performed even in the acute states.

Length of the therapy/follow-up was not the same for each group. In patients with acute back pain (Group 1), the time between two visits was usually shorter (according to clinical findings in the back or the leg) than in patients with chronic pain. Average therapy duration was 10 weeks. In Group 2, the interval between check-ups was usually longer than in Group 1 because the participants of this group had no clinical symptoms. Average therapy duration was 20 weeks. It was necessary to visit and treat patients with pain (from Group 1) manually and also to actualize individual exercises earlier than for participants from Group 2 (who were without pain). Time of follow-up in Group 3 was planned in between times for Groups 1 and 2. Follow-up was planned at 15 weeks (based on the MEIS system setup recommendations), but in reality, based on the discipline and circumstances of the patients, it was 14 weeks.

### 2.3. H Score

H Score parameter was the assessment tool of MEIS CK therapy efficacy. H Score was defined from the Total Dysfunction parameter (TD) and the presence/absence of the symptoms (lower back pain). TD is a useful marker for therapy effectiveness, and it expresses the overall sum of functional problems of the locomotor system (especially postural and biomechanical ones). Total Dysfunction is a single numerical value that shows the sum of the failures of the locomotor system, especially the posture, and the biomechanical ratios. The MEIS CK software evaluates the functional relationships of the right and left body parts in the frontal plane, the sagittal functional relations in the anterior-posterior plane, and the horizontal relations between the upper and lower body parts. The Total Dysfunction colored band graph was based on the results of the abovementioned tests and individual test results (degree of examination: A, B, or C). The TD parameter was divided into 4 different color zones (yellow, ≤59; green, 60-119; blue, 120-179; and red, ≥180-240). Three consecutive examinations were needed to evaluate the effectiveness of therapy over the course of days or weeks.

**Figure 1:**
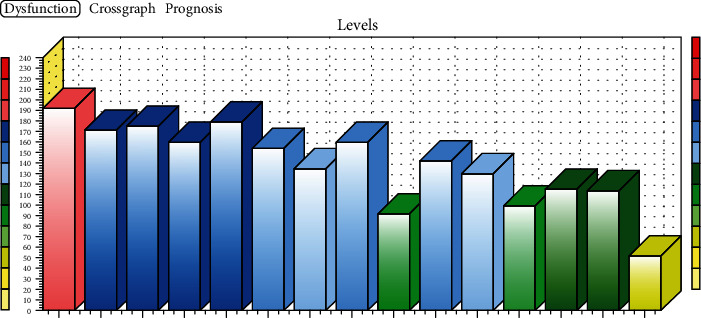
Illustrative graph of TD (an example of improving a person treated with MEIS CK therapy).

In the yellow zone (zone of ideal values), there can be persons who had almost all the test results within degree of examination A (normal function). Upon entering the study, there were no participants in the yellow zone. In the green zone, lighter functional disorders were present and tests were rated mostly A and sometimes B, and for exceptional cases, C. In the blue zone, which is represented by healthy people with functional deficits and minimal structural changes, there is a predominance of degree B and may contain degrees A and C. In the red zone, there are persons with most tests graded C (having more severe musculoskeletal disorders, e.g., acute problems and structural changes) (Figure 1).

H Score grades (0-3) were introduced because of the clinical significance of the study. The yellow zone is represented by H Score grade 0 (TD 0-59), the green zone is represented by H Score grade 1 (TD 60-119), the blue zone is represented by H Score grade 2 (TD 120-179), and the red zone is represented by H Score grade 3 (TD 180-240).

### 2.4. Statistical Analysis

An overview of demographic variables at baseline was presented using descriptive statistics and their 95% confidence intervals. For the baseline values of these criteria, the parametric test (Student's *t*-test or one-way ANOVA) was used for continuous variables and the Fisher's exact or chi-square tests were applied for dichotomous variables.

The improvement rate (IR), defined by at least a one-point decrease of the H Score (improvement in one color zone of TD (see paragraph above) that corresponds to a decrease by one H Score grade (one color zone), was adopted as the primary endpoint. This score was established for the purpose of this study, and it was composed of an objective parameter (TD, obtained from the MEIS CK) and a subjective parameter (absent or present back pain in subject).

The primary objective of the study was to confirm that the improvement rate (IR) of Group 1 (p) was not worse than Group 2 (h). The sample size was derived from the null hypothesis that the difference in the improvement rate between Group 1 (p) and healthy subjects was equal to or lower than -20%, i.e., IR_p_ − IR_h_ ≤ −20%, including the lower limit of the 95% confidence interval at the probability of a type 1 error of 0.05 at the statistical power of 80%. The number of subjects achieved at least 50 for each group to demonstrate the aim of the study.

Effect size was considered as an “effect of the time change” with *p* values of effect sizes between Groups 1, 2, and 3 included in the last row of Table 2.

The sample size of 173 subjects was justified for logistic regression using 5 covariates according to Peduzzi et al. [49]. McFadden's *R*-squared approach was higher than 0.4 indicating a good predictive ability of this model for the selected predictors. The continuous variables of predictors were assessed according to the median of the entire study population. The association was evaluated with the odds ratio mutually adjusted for all selected predictors, including a 95% confidence interval.

All tests were two-tailed, and the level of significance was set at 0.05. Statistical analyses and logistic regression were performed with Prism 8 (GraphPad Software, Inc., La Jolla, California, USA) and STATA version 15.1 software (StatCorp, Lakeway Drive, Texas, USA), respectively.

## 3. Results

The average TD in all three groups before and after the therapy/follow-up is shown in Table 2. We found significant improvement of dysfunction in Groups 1 and 2 after therapy/follow-up. The average TD remained different in all three groups before the therapy/follow-up (*p* = 0.0003), and after (*p* < 0.0001). Moreover, there was a statistically significant difference between Groups 1 and 2 before (*p* = 0.0023), and after the therapy (*p* = 0.0096).

**Figure 2:**
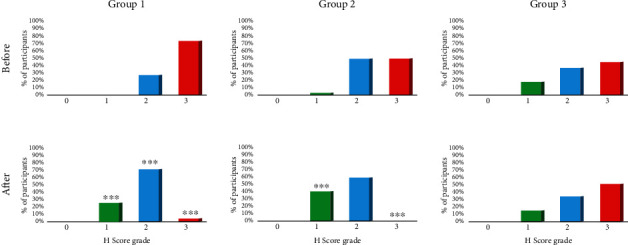
Proportion of H Score before and after CK therapy (Group 1, Group 2)/follow-up (Group 3), ^∗∗∗^*p* < 0.0001.

The distribution of H Score in all three groups before and after the therapy/follow-up is shown in Figure 2. A significant improvement of H Score grade was generally observed in Groups 1 and 2. The distribution of H Score in Group 1 before the therapy was grades 2 and 3; after the therapy, there was a rearrangement to grades 1 (*p* < 0.0001), 2 (*p* < 0.0001), and 3 (*p* < 0.0001). Out of 40 patients in grade 3 before the therapy, only 2 remained within the same grade after the therapy; a significant improvement was even observed in 17 patients after therapy, so that they moved to grades 1 and 2, respectively. Group 2 had H Score grades of 1, 2, and 3 before the therapy, but after the therapy, it only had grades 1 (*p* < 0.0001) and 2 (*p* = 0.4270); out of 25 participants who were formerly in grade 3, none remained within grade 3 after the therapy (*p* < 0.0001). In Group 3, H Score grades before the follow-up were 1, 2, and 3; after the follow-up, the grades were still 1 (*p* = 0.8161), 2 (*p* = 0.8571), and 3 (*p* = 0.6040). Obviously, in Group 1, there was a rearrangement from a higher H Score grade to a lower one. Similarly, the same thing happened in Group 2. There was no change of distribution of H Score grade in Group 3 (Figure 2).

Table 3 documents that the improvement rate was 87.3% (95% Cl: 75.5 to 94.7%) for Group 1 and 78.4% (95% Cl: 64.7 to 88.7%) for Group 2. The primary objective of this study was achieved because the difference of improvement rates between Group 1 and Group 2 reached 8.8% (95% Cl: -5.5 to 23.2%), and therefore, the null hypothesis had to be rejected. The CK therapy in Group 1 was not worse than that in Group 2.

Furthermore, the 11.9% improvement rate in Group 3 confirmed the superiority of both groups with CK therapy; the difference of the IR was 75.3% (95% Cl: 63.6 to 87.1%) between Group 1 and Group 3 and 66.5% (95% Cl: 52.8 to 80.2%) between Group 2 and Group 3.

Results in Table 4 confirmed that the other factors (gender, age, and BMI) have not influenced the result of the CK therapy.

## 4. Discussion

Our pilot study offers verification of the efficiency of the MEIS CK method in acute and chronic low back pain. In our study, 55 patients with acute and chronic back pain (secondary prevention) were treated, with an average treatment period of 71 days, and 51 healthy participants without LBP (primary prevention) were also treated, with an average treatment period of 141 days.

Treatment improvements in Groups 1 and 2 were due solely to the effect of the MEIS CK therapy. In Group 2, we treated 50% fewer men (*N* = 12) compared to Group 1 (*N* = 24) because women's interest in preventing back pain was higher.

We were successful in the improvement of conditions in 87% (48 of 55) of Group 1: 17 of 48 patients were completely deprived of back pain. In Group 1, redistribution of the H score from grade 3 to grades 1 and 2 seems to be a very important result—it means that participants were without symptoms (back pain) or functional disorders of the locomotor system were gone. Improvement in Group 2 was observed in 78.4% (i.e., 40 participants of the total number 51). Surprisingly, the H score of all 25 participants with grade 3 got better minimally by 1 grade. Participants from Group 2 with grades 1 and 2 were not patients with back pain, so MEIS CK could act as a primary means of prevention for back pain and a means of prevention for functional disorders of the locomotor system. Effect size analysis showed that treated Groups 1 and 2 had significant improvement in time.

MEIS CK is appropriate as a valid objectification tool in physiotherapy for early diagnostics and treatment of functional disorders of the motor system. Advantages of MEIS CK are as follows: standardization of tests, integral individual approach, and adequacy of the therapy considering the current condition of the patient.

Treatment of precisely selected muscles and soft tissues according to the MEIS CK algorithm helps to achieve fast reflexes and long-term therapeutic effects without adverse negative stress to the patient. Thanks to the visualization of actual findings in well-arranged charts, the patient is educated and motivated to performing everyday exercises selected individually by the MEIS CK software. Utilization of the diagnostic part of the MEIS CK system meets the WHO requirements for evidence by the EBM concept, and it can be used for the evaluation of the effectiveness of physiotherapeutic treatment procedures. MEIS CK is mostly used in outpatient practices of physiotherapists. It is also used in the frame of hospitalization in physiotherapeutic facilities and in the spa care [38–41].

Conservative treatment of back pain is subject to various physiotherapy methods worldwide. We failed to identify any single study that would deal with the treatment of low back pain similarly as the MEIS CK method, i.e., a combination of a manual correction of reflexive changes of soft tissues and physical exercise with a specific breathing regime in the second's rhythm. Next in Discussion, we present different methods that are more often used for the treatment of back pain. Unfortunately, we cannot compare our therapy results with the results of studies below.

Nonspecific low back pain is a large and costly problem. It has a lifetime prevalence in 80% of the workforce and is associated with high levels of fear avoidance and kinesiophobia. Although exercise is considered a modest effective treatment for chronic LBP, current evidence suggests that no single form of exercise is superior to another [25]. Nevertheless, stabilization exercises have been suggested to reduce symptoms of pain and disability and form an effective treatment. Meta-analysis showed the significant benefit of stabilization exercises versus any other treatment for control of long-term pain and disability with a mean difference of -6.39 (95% CI: -10.14 to -2.65) and -3.92 (95% CI: -7.25 to -0.59), respectively. There is strong evidence that stabilization exercises are not more effective than any other form of active exercise in the long term [25].

Among the other commonly used exercise interventions is motor control exercise (MCE). In 29 trials (*N* = 2431) with study samples ranging from 20 to 323 participants, pain intensity and disability were monitored as primary outcomes and function, quality of life, return to work, and recurrence were monitored as secondary outcomes. The overall results of the studies were that MCE is not superior to other forms of exercise and that the choice of exercise for chronic LBP should probably depend on patient or therapist preferences, therapist training, costs, and safety **[**50].

Motor control exercises to improve control and coordination of trunk muscles and graded activity under the principles of cognitive-behavioral therapy are examples of another 2 commonly used exercise therapies. The participants (172 patients) with chronic (>12 weeks) nonspecific LBP were randomly assigned to receive either motor control exercises or graded activity. Primary outcomes were average pain over the previous week (numeric rating scale) and function (Patient-Specific Functional Scale); secondary outcomes were disability (the 24-Item Roland-Morris Disability Questionnaire), global impression of change (Global Perceived Effect Scale), and quality of life (36-Item Short-Form Health Survey Questionnaire (SF-36)). Results of this study suggest that motor control exercises and graded activity have similar effects for patients with chronic nonspecific LBP [14].

The Back School consists of a therapeutic programme given to groups of people that includes both education and exercise. A study evaluating the effect of the Back School approach in patients with acute and subacute LBP came to the following conclusions: it is more effective than no treatment (MD: -6.10; 95% CI: -10.18 to -2.01); it has no difference at the intermediate term (MD: -4.34; 95% CI: -14.37 to 5.68) or long-term follow-up (MD: -12.16; 95% CI: -29.14 to 4.83); it reduces pain at a short-term follow-up compared to medical care (MD: -10.16; 95% CI: -19.11 to -1.22); and there are similarly many other instances showing that it has a questionable effect compared to other systems [51].

Concerning treatment effect, spinal manipulative therapy (SMT) is one of the many therapies for the treatment of LBP. There is a high-quality evidence that SMT has a small, significant, but not clinically relevant, short-term effect on pain relief (MD: -4.16; 95% CI: -6.97 to -1.36) and functional status (SMD: -0.22; 95% CI: -0.36 to -0.07) in comparison with other interventions. There is a very low-quality evidence that SMT has a significant short-term effect on pain relief and functional status when added to another intervention. Data were particularly sparse for recovery, return to work, quality of life, and costs of care. High-quality evidence suggests that there is no clinically relevant difference between SMT and other interventions for reducing pain and improving function in patients with chronic LBP [12].

McKenzie's method is the most widely supported physiotherapeutic method by numerous studies at present. Studies on McKenzie's method (e.g., [3, 52]) reported a greater decrease in pain and disability in the short-term follow-up, while was there was no documented difference between McKenzie's method and other standard treatments in the intermediate-term follow-up. This is a similar situation with that of a study focused on the results of the Back School mentioned above [51]. Unfortunately, data on the long-term effects of McKenzie's approach are not known yet. Also, it is mentioned in both studies that there is a lack of relevant data for McKenzie's method in patients with neck pain [3, 52].

Another review showed that for LBP patients, McKenzie's therapy does result in a greater decrease in pain and disability in the short term compared to other standard therapies. But making a firm conclusion on LBP treatment effectiveness is difficult because there are insufficient data on long-term effects on outcomes other than pain and disability, and no trial has yet compared McKenzie's method to placebo or no treatment. There are also insufficient data available on neck pain patients [3].

The first limitation of this study is the duration of treatment length: we were unable to perform three consecutive MEIS CK diagnostics and therapy at the same time intervals in all treated patients (Group 1) and participants (Group 2), because patients from Group 1 with LBP came for visits earlier than healthy participants from Group 2.

Subjective pain assessment using the Visual Analogue Scale (VAS) was not utilized because it was applicable in Group 1 only (Groups 2 and 3 were with no LBP). These patients had different lengths of pain duration, and chronic patients had different recurrence rates.

Finally, despite that smoking was not in the exclusion criteria, only four out of the total 173 persons admitted history of smoking in their anamnesis. For this reason, we could not evaluate the impact of smoking on the result of MEIS CK therapy, although its negative effect on the therapy is well recognized. Therefore, we have decided to neglect this factor in our study. In addition to physiotherapy, we also checked other factors (gender, age, and BMI) that could affect the outcome of therapy. It was shown that none of these factors influenced the outcome of MEIS CK therapy.

## 5. Conclusion

In our pilot study, we have focused on the therapy of acute and chronic back pain by Medical Expert Information System Computer Kinesiology. Patients with back pain did not respond to the conventional treatment prior to the onset of our therapy. Apart from the group of patients treated by physiotherapy, also a group without any pain underwent the CK therapy. To them, the therapy was a primary means of prevention for back pain. We also had a healthy control group. People in this group were only diagnosed and followed in time. The dissimilar length of the therapy in treated groups was caused by the presence or absence of symptoms (i.e., back pain or radicular pain in lower limb).

The effect of CK therapy was objectively assessed by the primary H Score parameter. H Score was derived from Total Dysfunction of the motor system and the presence or absence of symptoms at the beginning and end of therapy/follow-up. Subjective assessment of the efficacy of the treatment by VAS (Visual Analogue Scale) was not performed. Apart from physiotherapy, we focused also on checking other factors (gender, age, and BMI), but none of these factors have influenced the result of the therapy. The improvement in treated Groups 1 and 2 was caused exclusively by CK therapy.

On the basis of our study, we can postulate that Medical Expert Information System Computer Kinesiology may serve as the useful diagnostic tool for functional disorders that expands visualization methods alike X-ray, CT, MRI, and also other examinations.

## Figures and Tables

**Table 1:** Characteristics of participants in the study.

	Characteristics			
	Sex (M/F)	Age ± SD (years)	BMI ± SD (kg/m^2^)	Follow‐up ± SD (days)
Group 1	24/31	43.7 ± 9.7	26.4 ± 4.5	70.5 ± 51.7
Group 2	12/39	47.4 ± 13.5	25.6 ± 5.3	140.9 ± 90.3
Group 3	21/46	36.5 ± 15.7	25.0 ± 4.5	99.8 ± 65.4

**Table 2:** Mean total dysfunction.

	Groups			ANOVA	Student's *t*-test (*p*)
	Group 1	Group 2	Group 3		
TD before	190.8 ± 23.39	176.3 ± 24.29	167.8 ± 39.52	(*p*) 0.0003 (*F*) 8.417 (DF) 172	0.0023
TD after	134.6 ± 26.06	121.9 ± 23.03	173.4 ± 40.27	(*p*) < 0.0001 (*F*) 43.4 (DF) 172	0.0096
Effect size^∗^	56.2	54.4	-5.6	n/a	n/a
*p* (Student's *t*-test)	<0.0001	<0.0001	0.0420	n/a	n/a

^∗^The difference between TD before and TD after.

**Table 3:** Proportions and the gross and adjusted ratios of chances of improvement depending on group.

Predictor		*N*	*n*	Proportions (%)	cOR (95% CI)	aOR (95% CI)	*P* (LR)
Group	2	51	40	78.4 (64.7-88.7)	1.0	1.0	
	1	55	48	87.3 (75.5-94.7)	1.89 (0.67-5.32)	2.05 (0.65-6.43)	0.318
	3	67	8	11.9 (5.3-22.2)	0.04 (0.01-0.10)	0.04 (0.01-0.11)	<0.0001

*N*: total number in the group; *n*: number of improvements in the group, proportion of improvements; cOR: rough ratio of chances; aOR: adjusted ratio of chances; *P* (LR): *p* value determined by logistic regression.

**Table 4:** Proportions and the gross and adjusted ratios of chances of improvement depending on other factors.

Predictor		*N*	*n*	Proportions (%)	cOR (95% CI)	aOR (95% CI)	P (LR)
Sex	Male	57	35	61.4 (47.6-74.0)	1.0	1.0	
	Female	116	61	52.6 (43.1-61.9)	0.70 (0.37-1.33)	0.61 (0.22-1.67)	0.509
Age	<43.7 years	86	42	48.8 (37.9-59.9)	1.0	1.0	
	≥43.7 years	87	54	62.1 (51.0-72.3)	1.71 (0.94-3.14)	0.97 (0.38-2.47)	0.991
BMI	<24.3 kg/m^2^	85	44	51.8 (40.7-62.7)	1.0	1.0	
	≥24.3 kg/m^2^	88	52	59.1 (48.1-69.5)	1.35 (0.74-2.46)	0.90 (0.34-2.36)	0.716
Follow-up/therapy	<84 days	79	41	51.9 (40.4-63.3)	1.0	1.0	
	≥84 days	94	55	58.5 (47.9-68.6)	1.31 (0.72-2.39)	1.53 (0.61-3.85)	0.170

*N*: total number in the group; *n*: number of improvements in the group, proportion of improvements; cOR: rough ratio of chances; aOR: adjusted ratio of chances; *P* (LR): *p* value determined by logistic regression.

## Data Availability

The data (two figures and four tables) used to support the findings of this study are included within the article.
